# Influenza A Presenting as Life-Threatening Rhabdomyolysis and Acute Kidney Injury Requiring Renal Replacement Therapy

**DOI:** 10.7759/cureus.114111

**Published:** 2026-08-07

**Authors:** Sashwat R Lamichhane, Anand Thakur, Ayusha Thapa, Shirish R Joshi

**Affiliations:** 1 Intensive Care Unit, Grande International Hospital, Kathmandu, NPL; 2 Intensive Care Unit, National Academy of Medical Sciences, Kathmandu, NPL

**Keywords:** acute kidney injury, creatine kinase, hemodialysis, influenza a, rhabdomyolysis

## Abstract

Influenza A is primarily a respiratory pathogen but may rarely cause severe extrapulmonary manifestations, including myositis, rhabdomyolysis, and acute kidney injury (AKI). Recognition of this complication is often delayed because respiratory symptoms may be absent or overshadowed by systemic manifestations. We present a case of a previously healthy 31-year-old man who presented with fever, generalized myalgia, oliguria, and dark urine. Laboratory evaluation demonstrated extreme rhabdomyolysis with a peak creatine kinase (CK) concentration of 468,130 U/L, severe transaminitis consistent with severe skeletal muscle injury, myoglobinuria, electrolyte abnormalities, and rapidly progressive AKI. Extensive evaluation excluded alternative infectious and noninfectious etiologies. Respiratory viral polymerase chain reaction testing confirmed influenza A infection. Despite aggressive fluid resuscitation and metabolic correction, worsening renal dysfunction necessitated six sessions of intermittent hemodialysis. Following treatment with oseltamivir, renal replacement therapy, and supportive care, the patient experienced progressive biochemical recovery and was discharged without ongoing dialysis requirements. Influenza-associated rhabdomyolysis is uncommon in adults, and severe cases requiring renal replacement therapy remain rare. Proposed mechanisms include direct viral invasion of myocytes, cytokine-mediated muscle injury, and systemic inflammatory stress. This case was characterized by an exceptionally high CK concentration of 468,130 U/L. It highlights the potential for fulminant muscle injury even in the absence of prominent respiratory manifestations. Influenza A should be considered in patients presenting with unexplained rhabdomyolysis, myoglobinuria, and AKI during the influenza season. Early antiviral therapy, aggressive supportive care, and timely initiation of renal replacement therapy may be lifesaving.

## Introduction

Rhabdomyolysis refers to the breakdown of striated muscle, a syndrome characterized by muscle degradation and cell death, releasing intracellular constituents into the bloodstream and extracellular fluid [1]. It is commonly triggered by direct muscle injury, but may also result from enzyme deficiencies, electrolyte disturbances, infections, medications, toxins, or endocrine disorders. Myoglobinuria is a frequent accompanying finding, and severe cases can progress to acute renal failure [1]. External toxins, particularly illicit drugs, alcohol, and specific medications, remain the leading identifiable cause, although genetic myopathies, strenuous exercise, compartment syndrome, and inflammatory disease also contribute [2].

Among infectious causes, viral pathogens are an increasingly recognized but often overlooked trigger and may present atypically. COVID-19, for example, has been shown to precipitate autoimmune muscle injury (MDA5-associated necrotizing myositis) with severe rhabdomyolysis, even in the absence of typical pulmonary or cutaneous manifestations [3]. Influenza A, an RNA virus of the family Orthomyxoviridae, primarily causes respiratory illness but can rarely produce extrapulmonary complications, including rhabdomyolysis. The mechanism underlying influenza-associated rhabdomyolysis remains incompletely understood. Proposed pathways include direct viral invasion of muscle tissue, cytokine-mediated muscle injury, and viral toxin-related damage. Recent evidence indicates that the influenza A virus can directly infect and replicate within skeletal muscle cells, leading to myocyte lysis and rhabdomyolysis [4].

We describe a case of severe influenza A-induced rhabdomyolysis complicated by dialysis-requiring acute kidney injury (AKI), highlighting the importance of early recognition of this uncommon but potentially fatal complication.

## Case presentation

A 31-year-old previously healthy man presented with a three-day history of acute-onset fever with chills and rigors, followed by generalized myalgia and dark-colored urine for two days. He denied strenuous exercise, trauma, seizures, or exposure to drugs or toxins. He had traveled to a hilly region 15 days before symptom onset by routine road transportation. During the trip, he denied hiking, trekking, camping, strenuous physical activity, or any unusual physical exertion. Workup for tropical infections, including dengue, malaria, leptospirosis, and scrub typhus, was negative. He initially presented to a peripheral hospital, where investigations revealed severe rhabdomyolysis complicated by AKI with hyperkalemia. Despite initial management, he developed oliguria with worsening renal function and was referred to our tertiary care center.

**Figure 1:**
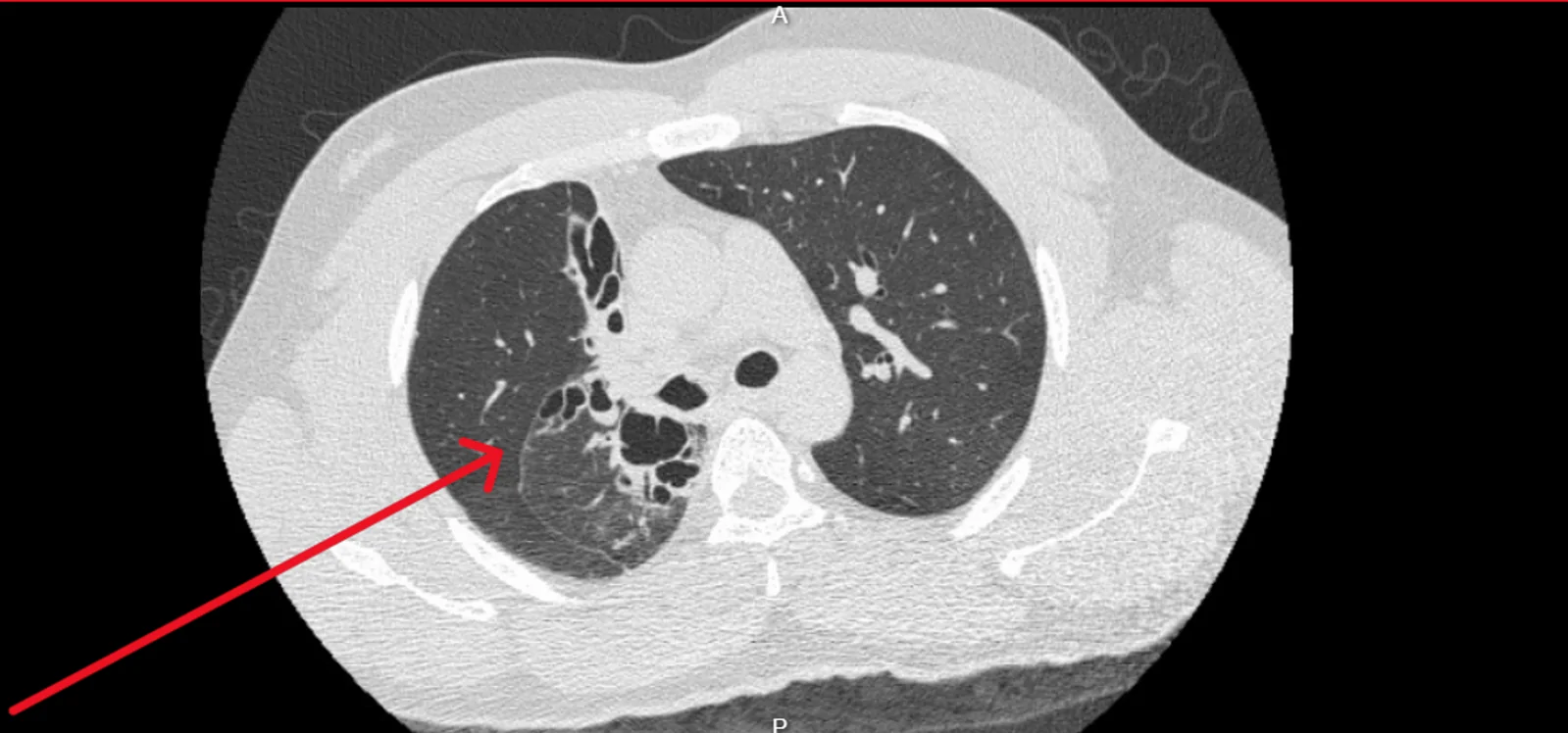
High-resolution CT of the chest demonstrating right-sided cystic bronchiectatic changes with superimposed patchy ground-glass opacities and centrilobular nodules, consistent with an acute infective process.

On admission, laboratory investigations revealed severe rhabdomyolysis, AKI, significant electrolyte disturbances, markedly elevated liver transaminases, and raised inflammatory markers, as shown in Table 1. Given the absence of evidence of primary liver disease, the elevated transaminases were considered to reflect skeletal muscle injury related to rhabdomyolysis. Urinalysis was positive for myoglobinuria. Further evaluation identified influenza A infection, confirmed by reverse transcription polymerase chain reaction (RT-PCR) from a respiratory viral panel in the intensive care unit (ICU). Following confirmation of influenza A by RT-PCR, a chest X-ray was done. It demonstrated patchy right lung air-space opacities with a few tram-track opacities, raising suspicion for an acute infective process. High-resolution chest CT (HRCT) was subsequently performed for further evaluation. It showed right-sided bronchiectatic changes with superimposed patchy ground-glass opacities and centrilobular nodules, suggestive of an acute infective process, as shown in Figure 1. Echocardiography demonstrated normal biventricular function.

**Table 1:** Baseline laboratory investigations on admission.

Parameter	Unit	Admission value	Reference range
Creatine kinase	U/L	418,000	39–308
Serum creatinine	mg/dL	2.7	0.7–1.3
Blood urea	mg/dL	83	15–45
Serum sodium	mmol/L	120	135–145
Serum potassium	mmol/L	6.22	3.5–5.1
Total serum calcium	mg/dL	6.0	8.8–10.1
Serum phosphorus	mg/dL	14.0	2.5–4.5
Serum albumin	g/dL	2.4	3.4–5
Aspartate aminotransferase	U/L	4,909	10–40
Alanine aminotransferase	U/L	1,013	7–56
Lactate dehydrogenase	U/L	35,372	135–225
Total leukocyte count	×10⁹/L	30.0	4.0–11.0
C-reactive protein	mg/L	151	<5

The patient was managed in the ICU with aggressive intravenous hydration, urine alkalinization, correction of electrolyte abnormalities, empirical antimicrobials, N-acetylcysteine, and supportive care. Empirical antimicrobial therapy included intravenous ceftriaxone and azithromycin. Oseltamivir was initiated as antiviral therapy. Due to worsening renal function and refractory metabolic derangements, he required a total of six hemodialysis sessions. The first hemodialysis session was initiated on the second day of ICU admission. The patient’s clinical course is summarized chronologically in Table 2.

**Table 2:** Chronological timeline of clinical events, investigations, and management. Creatine kinase reference range: 39–308 U/L; creatinine reference range: 0.7–1.3 mg/dL. AKI = acute kidney injury; CK = creatine kinase; HRCT = high-resolution computed tomography; ICU = intensive care unit; RT-PCR = reverse transcription polymerase chain reaction

Timepoint	Event
Day −15	Travel to a hilly area; no strenuous exertion reported
Day 1–3 (before admission)	Acute-onset fever with chills/rigors, followed by generalized myalgia and dark-colored urine
Presentation (peripheral hospital)	Severe rhabdomyolysis with AKI and hyperkalemia identified; oliguria and worsening renal function despite initial management; referred to a tertiary center
ICU day 1 (tertiary admission)	Peak biochemical derangement; CK 418,000 U/L; creatinine 2.7 mg/dL; hyperkalemia and hyponatremia; tropical infection panel negative; RT-PCR confirms influenza A
ICU day 2	CK peaks at 468,130 U/L; oseltamivir initiated; first hemodialysis session
ICU day 2–8	A total of six hemodialysis sessions for refractory metabolic derangement; HRCT chest performed, echocardiography normal
ICU day 8–10	Progressive decline in CK and transaminases; renal parameters stabilize; stepped down from intensive care
Post-ICU	Continued clinical and biochemical improvement; discharged home

**Figure 2:**
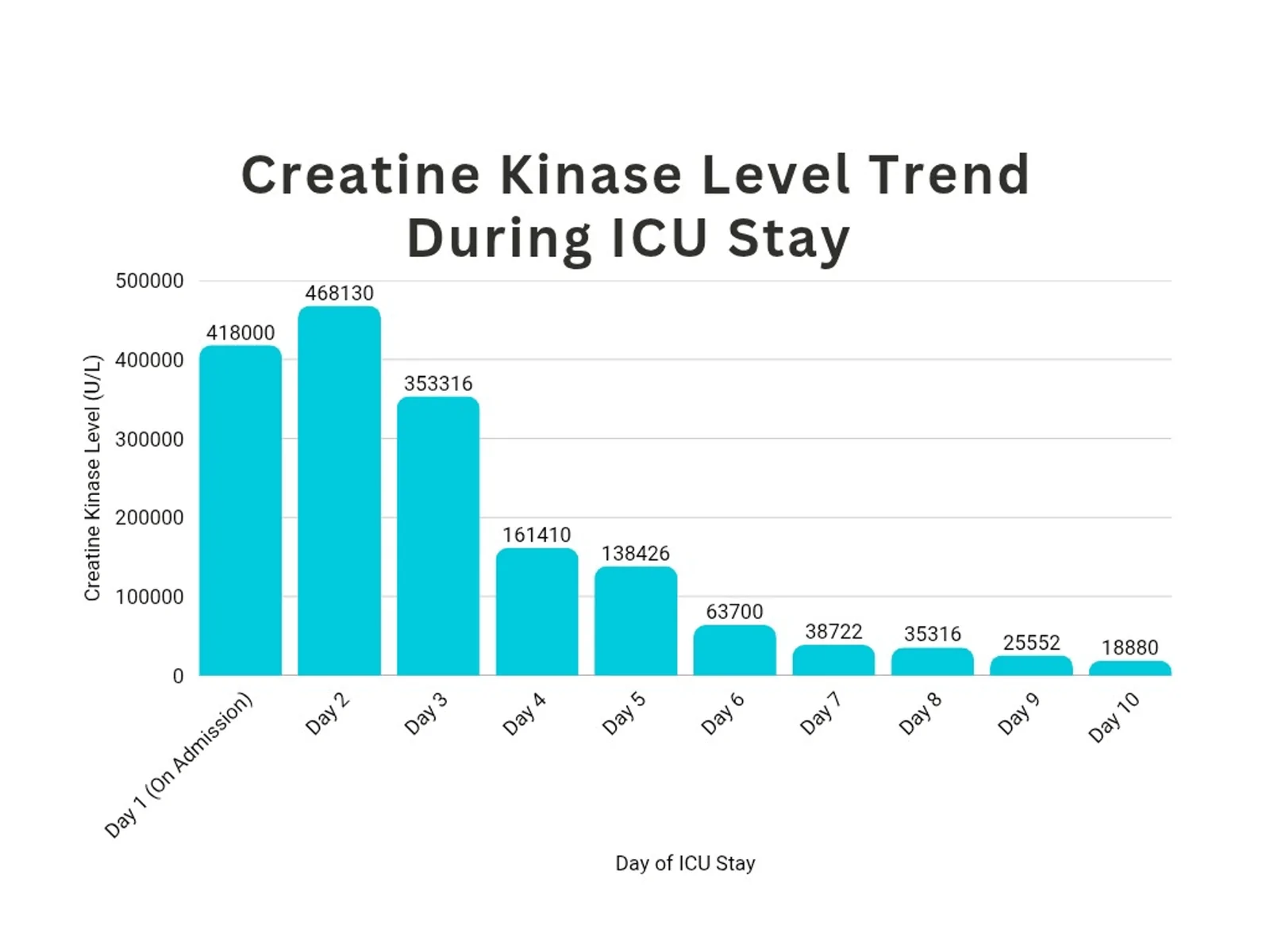
Serial changes in serum creatine kinase levels during the ICU course. ICU = intensive care unit

He demonstrated gradual clinical and biochemical improvement, with declining creatine kinase (CK) levels, as shown in Figure 2. During the ICU stay, there was a gradual improvement in the biochemical parameters, as shown in Table 3. Following this, he was stepped down from intensive care. After ICU discharge, he showed progressive clinical and biochemical improvement and was subsequently discharged home.

**Table 3:** Serial laboratory parameters during the intensive care unit course.

Laboratory parameter	Reference range	Day 1 (admission)	Day 2	Day 3	Day 4	Day 5	Day 6	Day 7	Day 8	Day 9	Day 10
Creatine kinase (U/L)	39–308	418,000	468,130	353,316	161,410	138,426	63,700	38,722	35,316	25,552	18,880
Creatinine (mg/dL)	0.7–1.3	2.7	3.2	5.0	4.9	4.8	4.4	4.0	5.9	4.7	4.9
Urea (mg/dL)	15–45	83	97	134	93	93	81	78	139	98	93
Sodium (mmol/L)	135–145	120	124	131	135	138	136	137	140	133	135
Potassium (mmol/L)	3.5–5.1	6.22	4.31	5.50	4.13	5.00	4.70	4.65	4.80	4.20	4.10
Aspartate aminotransferase (U/L)	10–40	4,909	4,805	4,741	2,950	1,675	966	609	440	200	155
Alanine aminotransferase (U/L)	7–56	1,013	1,026	1,088	880	629	509	400	356	155	98

## Discussion

This case highlights a severe, life-threatening presentation of influenza A-associated rhabdomyolysis complicated by dialysis-dependent AKI. With no obvious precipitant identified at presentation, the markedly elevated CK level was a key diagnostic challenge for the treating team.

Rhabdomyolysis is a potentially life-threatening condition characterized by skeletal muscle breakdown and release of intracellular contents, including myoglobin, CK, potassium, and phosphate, into the circulation. It can deteriorate rapidly and is fatal if not promptly identified and treated [5]. The clinical spectrum ranges from asymptomatic CK elevation to severe disease, often complicated by electrolyte imbalance, AKI, and multi-organ dysfunction [1,2]. Although toxins, trauma, drugs, and exercise are the most common causes of rhabdomyolysis, viral infections such as influenza A appear to be a rare but important trigger of fulminant disease [6]. Other viral agents, including enterovirus and Epstein-Barr virus, have similarly been associated with presentations ranging from mild muscle injury to severe rhabdomyolysis with systemic complications [7].

Several mechanisms have been proposed for influenza A-related rhabdomyolysis, including direct viral invasion and replication within muscle cells leading to lysis and spread to adjacent cells [4], and release of pro-inflammatory cytokines such as interleukin-6 (IL-6) and tumor necrosis factor-alpha (TNF-α) that mediate immune-related muscle injury [6]. Systemic inflammatory response and metabolic stress during infection, as well as viral toxin production, may further contribute to muscle injury [6]. Marked CK elevations, as observed in this patient (468,130 U/L), are closely associated with an increased risk of AKI [8]. Myoglobin-induced tubular toxicity, renal vasoconstriction, and intravascular volume depletion together drive acute renal failure and pigment nephropathy, with the severity of renal injury influenced by dehydration, delayed presentation, and the extent of muscle breakdown [8,9]. Influenza-associated rhabdomyolysis has been reported across a range of clinical contexts and age groups, including severe systemic influenza and pandemic strains such as H1N1 [9,10].

Dialysis-dependent AKI remains relatively uncommon, but severely affected patients may require critical care support and renal replacement therapy. Viral-induced muscle injury may be compounded by systemic stressors such as fever, dehydration, and metabolic derangement; this “multi-hit” model proposes that inflammatory stress from illness lowers the threshold for muscle breakdown, particularly when combined with additional physiological demand [11].

In patients presenting with fever, severe myalgia, dark urine, and markedly elevated CK, a broad differential should be considered, including bacterial sepsis-related myopathy and other infectious myopathies such as dengue, leptospirosis, Epstein-Barr virus infection, and enteroviral disease. Non-infectious causes such as metabolic myopathies, autoimmune inflammatory myositis, drug- or toxin-induced muscle injury (including statins, alcohol, and illicit substances), and exertional rhabdomyolysis must also be excluded.

In this case, there was no history of trauma, strenuous activity, drug or toxin exposure, or clinical features of autoimmune disease. Influenza A infection, confirmed by RT-PCR, was determined to be the most likely precipitant based on laboratory and clinical findings, further supported by a negative tropical infection workup for dengue, malaria, leptospirosis, and scrub typhus. Despite fulminant rhabdomyolysis, significant biochemical derangement, and rapidly worsening AKI necessitating hemodialysis, the patient showed consistent clinical improvement. Although CK elevations of this magnitude with dialysis dependence are uncommon, severe influenza infection is associated with a broad spectrum of extrapulmonary complications that may necessitate intensive care management [10]. Renal function and urine output improved progressively, and CK levels declined with hemodialysis, vigorous fluid resuscitation, correction of metabolic imbalances, and oseltamivir therapy, allowing eventual weaning from intensive care.

Influenza A-associated rhabdomyolysis can vary considerably in severity, as shown in Table 4. Previously reported cases demonstrated peak CK levels ranging from 34,176 U/L to 412,000 U/L, with all patients developing acute kidney injury and recovering with supportive management. Our patient had the highest peak CK level (468,130 U/L) and was the only case among those compared to require hemodialysis for dialysis-dependent AKI [4-6]. Despite the severity of the presentation, the patient recovered completely following antiviral therapy, aggressive supportive care, and six sessions of hemodialysis, underscoring the importance of early recognition and timely management.

**Table 4:** Comparison of published cases of influenza A-associated rhabdomyolysis with acute kidney injury and the present case. AKI = acute kidney injury; CK = creatine kinase (39–308 U/L)

Study	CK peak (U/L)	AKI	Dialysis	Outcome
Fadila et al. [4]	34,176	Yes	No	Recovery
Runnstrom et al. [5]	76,015	Yes	No	Recovery
Velho et al. [6]	412,000	Yes	No	Recovery
Present case	468,130	Yes	Yes	Recovery

Early recognition and aggressive management remain the mainstay of treatment. Kidney replacement therapy is indicated for refractory electrolyte imbalance, severe acidosis, or uremia, while prompt and adequate intravenous fluid resuscitation is central to preventing myoglobin-induced kidney injury [1,2,8]. Early diagnosis of influenza infection may allow timely initiation of antiviral therapy. Oseltamivir, a neuraminidase inhibitor, was used in this case. Genetic predisposition should be considered in patients with severe or recurrent rhabdomyolysis. Disorders such as carnitine palmitoyltransferase II deficiency and RYR1-associated myopathies may increase susceptibility to muscle injury following infection or other physiological stressors [12].

However, genetic testing for underlying metabolic or hereditary muscle disease was not performed in our case. Such evaluation is generally reserved for patients with prolonged muscle weakness, atypical presentations, or recurrent rhabdomyolysis. As our patient showed steady clinical improvement and declining CK levels with gradual weaning from intensive care, additional invasive testing was not prioritized.

## Conclusions

Influenza A is usually a self-limiting respiratory illness, but can rarely lead to severe complications such as rhabdomyolysis and AKI. Early recognition is essential, particularly in patients presenting with muscle pain, dark urine, and markedly elevated CK levels. Prompt antiviral therapy, combined with aggressive supportive care, including hydration and renal replacement therapy when indicated, remains the cornerstone of management. This case underscores the importance of considering influenza-associated rhabdomyolysis as an uncommon but serious and potentially reversible complication.

## References

[1] Huerta-Alardín AL, Varon J, Marik PE (2005). Bench-to-bedside review: rhabdomyolysis -- an overview for clinicians. Crit Care.

[2] Melli G, Chaudhry V, Cornblath DR (2005). Rhabdomyolysis: an evaluation of 475 hospitalized patients. Medicine (Baltimore).

[3] Steadman S, Sikder A, Desai HR, Collins B, Baker T, Worthington J (2025). COVID-19-associated MDA5-mediated necrotizing myositis. Am J Case Rep.

[4] Fadila MF, Wool KJ (2015). Rhabdomyolysis secondary to influenza a infection: a case report and review of the literature. N Am J Med Sci.

[5] Runnstrom M, Ebied AM, Khoury AP, Reddy R (2018). Influenza-induced rhabdomyolysis. BMJ Case Rep.

[6] Velho R, Maia M, Belchior B (2026). Extreme rhabdomyolysis secondary to influenza A infection. Cureus.

[7] Tuchinsky A, Montalvo A, Lent D, Goldman J (2023). Acute myositis secondary to Epstein-Barr virus in the absence of infectious mononucleosis with severe rhabdomyolysis. BMJ Case Rep.

[8] Dye M, Lantz R (2024). Profound rhabdomyolysis and viral myositis due to SARS-CoV-2: a case report. Cureus.

[9] Chien SJ, Hsieh YJ, Shih YL, Tseng YJ (2022). Clinical characteristics and outcomes of mixed virus or bacterial infection in children with laboratory-confirmed influenza infection. J Formos Med Assoc.

[10] Kalil AC, Thomas PG (2019). Influenza virus-related critical illness: pathophysiology and epidemiology. Crit Care.

[11] Benaini I, Bouayed MZ, Oujidi Y, Ismaili MF, Laaribi I, Zaid I, Bkiyar H (2026). Severe rhabdomyolysis with acute kidney injury triggered by influenza A and strenuous exercise in a healthy adolescent: a dual-hit mechanism. Cureus.

[12] Scalco RS, Gardiner AR, Pitceathly RD (2015). Rhabdomyolysis: a genetic perspective. Orphanet J Rare Dis.

